# Cardiovascular Magnetic Resonance in patients with repaired Tetralogy of Fallot: the goal standard in preoperative assessment and follow up of injectable pulmonary valve implantation

**DOI:** 10.1186/1532-429X-15-S1-E86

**Published:** 2013-01-30

**Authors:** Aurelio Secinaro, Benedetta Leonardi, Stefano M Marianeschi, Antonio Amodeo, Carmela Napolitano, Fabrizio Gandolfo, Valentina Silvestri, Giacomo Pongiglione, Paolo Tomà

**Affiliations:** 1 Imaging, Bambino Gesù Pediatric Hospital, Rome, Italy; 2 Pediatric Cardiology and Cardiac Surgery, Bambino Gesù Pediatric Hospital, Rome, Italy; 3 Cardiothoracic Surgery, Niguarda Ca' Granda Hospital, Milan, Italy

## Background

Severe pulmonary regurgitation, progressive dilatation and dysfunction of the right ventricle are the most frequent reason of late morbidity post Fallot repair. Pulmonary valve replacement is often indicated in these patients. BioIntegral Injectable pulmonary valve (IPV) is an innovative less invasive technique, often done off cardiopulmonary bypass (CPB). Cardiovascular Magnetic Resonance (CMR) is fundamental tool to assess patient suitability for IPV insertion and in the clinical follow up.

## Methods

From January 2006 to June 2012, 10 patients undergoing a pulmonary valve replacement due to end-diastolic volume overload and pulmonary regurgitation (PR) assessed at CMR pre-surgery examination underwent IPV insertion.

We performed a full CMR (Achieva, 1.5 Tesla Philips) examination including right ventricular volume measurements, velocity encoded sequences and 3DSSFP ecg and respiratory gated with a navigator tecnique to asses pulmonary artery diameters.

In particular we measured pulmonary diameters in systole at the following sites: at the level of right ventricular patch, at the pulmonary valve and at the bifurcation and the length of the pulmonary trunk.

Suitability for IPV approach included a pulmonary trunk length more than 20 mm and a trunk diameter between 15 and 31 mm, due to the dimension of the biointegral valve.

For more than 31 mm diameter a pulmonary arterioplasty was needed.

5 of these patients underwent also a CMR 3 months to 6 years follow-up.

## Results

IPV was successful and with an excellent hemodynamic performance in all 10 patients.

Only 3 patients underwent concomitant procedures on CPB and no-one underwent reduction plasty of a dilated main pulmonary artery.

Early recovery was uneventful. There were no reoperations.

At the latest follow-up 3 months to 6 years post IPV insertion CMR showed an improvement of right ventricle end diastolic volume. The IPV was continent in the majority of the patients with no flow acceleration measured. We just reported a case of paraprosthetic mild to moderate PR.

## Conclusions

CMR is a safe and effective method to asses patient suitability for IPV insertion.

In the follow up CMR measure the pulmonary valve efficiency, the transvalvular gradients and the right ventricle function.

IPV is also better detected by CMR than traditional prosthesis valve that present focal artefacts that can obscure small jets.

## Funding

Nothing to declare.

**Figure 1:**
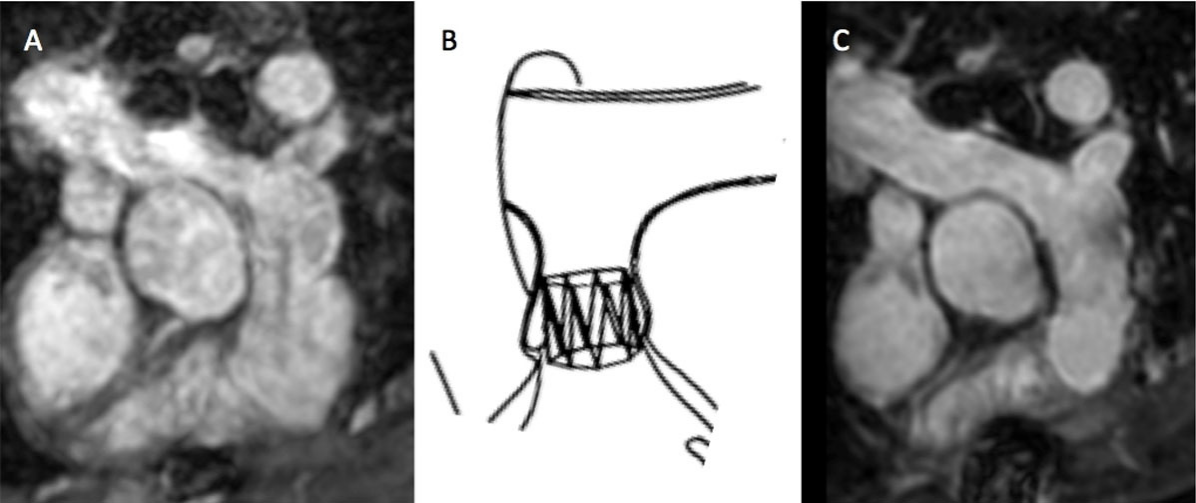
3D Whole-heart imaging of the MPA pre (A) and post (C) IPV implantation shows well seated device according to the draft (B) with low magnetic susceptibility of the device and adequate intra-stent visualization (no need for furter investigation such as CT or catheter).

**Figure 2:**
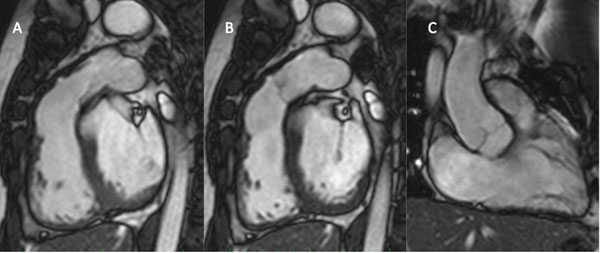
Balanced SSFP cine imaging of the RVOT post IPV implantation in systole (A) and diastole (B) allows good functional assessment of the valve leaflets. This is comparable to native valves such as aorta and tricuspid, as showed in the RV in and out view (C)

